# Draft Genome Sequence of *Pseudoalteromonas* sp. Strain A2, an Isolate with High Antioxidative Activity from Arctic Seawater

**DOI:** 10.1128/genomeA.00885-14

**Published:** 2014-09-11

**Authors:** Xuezheng Lin, Zhen Wang, Yang Li

**Affiliations:** Key Laboratory of Marine Bioactive Substances, First Institute of Oceanography, State Oceanic Administration, Qingdao, China

## Abstract

Here we report the draft genome sequence of *Pseudoalteromonas* strain A2, isolated from Arctic seawater in the pack-ice zone, which has high antioxidative activity against H_2_O_2_. The genomics information of this strain will facilitate the study of antioxidative mechanisms, cold adaptation properties, and evolution of this genus.

## GENOME ANNOUNCEMENT

The genus *Pseudoalteromonas* is one of the largest genera within the class *Gammaproteobacteria* and currently comprises more than 30 species. It was described to accommodate nonpigmented, Gram-negative, heterotrophic, aerobic, polar-flagellated species of marine bacteria that have DNA G+C contents ranging from 38 to 50 mol% (1). In recent years, novel species belonging to *Pseudoalteromonas* have been isolated from a wide range of habitats, including seawater (2, 3), marine sediment (4), tidal flat (5), and sponge (6). *Pseudoalteromonas* sp. strain A2 was isolated from Arctic seawater in the pack-ice zone (120°21.017′ E, 86°48.196′ N), collected during the 5th Chinese National Arctic Expedition, and it has high antioxidative activity against H_2_O_2_.

The genome of *Pseudoalteromonas* sp. strain A2 was sequenced with Illumina HiSeq 2000 platforms by Shanghai Majorbio Bio-pharm Technology Co., Ltd. (Shanghai, China). A paired-end library with a fragment length of 300–500 bp and a mate-pair library with a fragment length of approximately 3 kb were constructed, generating a total of 3,563,169 and 2,851,727 high-quality paired reads and representing 159-fold and 127-fold coverage, respectively. SOAPdenovo version 1.05 (http://soap.genomics.org.cn) was used to assemble the genome with multiple k-mer parameters. GapCloser software (from the SOAPdenovo website) was subsequently applied to fill the remaining local inner gaps and correct the single-base polymorphism for the final assembly result. Glimmer version 3.0 (7), tRNAscan-SE (8), and RNAmmer (9) were used to predict protein-encoding genes (CDSs), tRNAs, and rRNAs, respectively. Then all gene models were blasted against the NCBI nonredundant (NR), Swiss-Prot (http://uniprot.org), KEGG (http://www.genome.jp/kegg), and Clusters of Orthologous Groups (COG; http://www.ncbi.nlm.nih.gov/COG) databases for functional annotation.

The draft genome of *Pseudoalteromonas* sp. strain A2 comprises 4,170,731 bp, with an average G+C content of 40.13%, consisting of 59 contigs (contig *N*_50_, 214,730 bp). The genome sequence contains 3,703 candidate CDSs, and the average gene length is 984 bp. In addition, 161 tRNA genes for 20 amino acids and a 16S rRNA operon were identified in the genome. The genome sequence enables the further study of the antioxidative mechanisms, cold adaptation properties, and evolution of this genus.

### Nucleotide sequence accession numbers.

This whole-genome shotgun project has been deposited at DDBJ/EMBL/GenBank under the accession number JPMC00000000. The version described in this paper is version JPMC01000000.
